# First detection and phylogenetic analysis of porcine parainfluenza virus 1 and swine orthopneumovirus in swine reared on commercial farms in Serbia

**DOI:** 10.2478/jvetres-2026-0048

**Published:** 2026-08-24

**Authors:** Jelena Maksimović Zorić, Jelena Maletić, Dimitrije Glišić, Vladimir Radosavljević, Nemanja Zdravković, Nemanja Jezdimirović, Branislav Kureljušić

**Affiliations:** 1 Department of Virology, Institute of Veterinary Medicine of Serbia, 11000 Belgrade, Serbia; 2 Department of Epizootiology and Health Care of Poultry and Birds, Institute of Veterinary Medicine of Serbia, 11000 Belgrade, Serbia; 3 Department of Bacteriology and Parasitology, Institute of Veterinary Medicine of Serbia, 11000 Belgrade, Serbia; 4 Department of Epizootiology, Clinical Pathology, Pathological Morphology and Reproduction, Institute of Veterinary Medicine of Serbia, 11000 Belgrade, Serbia

**Keywords:** phylogenetic analysis, porcine parainfluenza virus 1, prevalence, Serbia, swine orthopneumovirus

## Abstract

**Introduction:**

Porcine parainfluenza virus 1 (PPIV1) and swine orthopneumovirus (SOV) are recently discovered respiratory viruses of swine. Data on their geographic distribution and genetic diversity are scarce. This study investigated the presence of these viruses in diseased pigs on commercial farms in Serbia and also the phylogenetic characteristics of the detected strains.

**Material and Methods:**

A total of 131 samples were collected during passive surveillance of swine influenza in pigs from 10 commercial farms. All samples were screened by real-time RT-PCR for swine influenza A virus (SIV) and PPIV1, and 77 of them were tested for SOV. Positive samples were further subjected to conventional RT-PCR targeting the PPIV1 *F* and SOV *G* genes, sequencing, and phylogenetic analysis.

**Results:**

Farm-level prevalence was 60% for SIV, 50% for PPIV1 and 10% for SOV. Overall, 27 samples were positive for SIV, 18 for PPIV1 and 2 for SOV. Co-circulation of two viruses was evident on four farms, and on one farm, all the tested viruses were detected. Phylogenetically, three Serbian PPIV1 strains clustered together with European and Asian strains, and SOV strains grouped with swine, murine and canine pneumoviruses, confirming their close evolutionary relationship.

**Conclusion:**

This is the first evidence of PPIV1 and SOV circulation on pig farms in Serbia. Even established phylogenetic patterns are consistent with those reported in Europe, expanded surveillance is needed to better define the prevalence and genetic diversity of these emerging respiratory viruses in Serbian pig populations.

## Introduction

*Respirovirus suis,* also known as porcine parainfluenza virus 1 (PPIV1), is a recently characterised virus that, according to the International Committee on Taxonomy of Viruses belongs to the subfamily Feraresvirinae and family Paramyxoviridae. The genome of this virus is single-stranded, negativesense RNA composed of six open reading frames, which encode six viral proteins: nucleocapsid, phosphoprotein, matrix, fusion (F), haemagglutinin-neuraminidase (HN), and large polymerase protein (35). As they are located on the surface of the virus and possess neutralising epitopes important for virus attachment and penetration into the cell, the HN and F proteins are commonly used for evolutionary analyses (6, 21, 33). The first detection and characterisation of PPIV1 was performed in Hong Kong in 2013, within a retrospective study of samples from pig carcasses collected from slaughterhouses between 2008 and 2012 (15). Its widespread distribution was later confirmed in other parts of Asia (12, 38), as well as in pig populations in the USA (21), Chile (1) and several European countries (6,10, 19, 29, 31, 37). Based on the full genome sequences of the fusion (*F*) gene, two distinct clades diverge: one encompassing the majority of European strains, and another representing American strains (33). According to available data, Chinese strains diverge into both clades (33, 38). This virus was confirmed in healthy pigs (6), pigs with mild respiratory signs, and pigs infected with other respiratory pathogens (29, 31, 36), but its influence on the pathogenesis of respiratory disorders was not fully understood. Experimental studies revealed its replication in the epithelium of the upper and lower parts of the respiratory tract (9), and its inhibitory effect on the production of interferon β and interferon-stimulating genes (16).

Swine orthopneumovirus (SOV) is a rarely detected swine virus that belongs to the *Orthopneumovirus* genus within the family Pneumoviridae (26). This virus is enveloped and possesses a negative-sense, unsegmented, single-stranded RNA genome consisting of 10 genes (26). Circulation of this virus in the swine population was first revealed in 1998 by the detection of swine antibodies reactive against bovine respiratory syncytial virus, another virus from the same genus (2). The first confirmation in pigs was achieved by metagenomic sequencing of feral swine nasal swabs in the USA, with the novel virus showing 90% nucleotide identity with canine pneumovirus (9). Serological investigation of antibodies against the nucleoprotein of SOV in French pigs revealed positivity among all tested herds, irrespective of the status of other respiratory pathogens (25). Circulation of the virus in nursery pigs with respiratory signs was further confirmed in Spain and Portugal, with a prevalence of 33.8% at the individual level (19). Detection of this virus in swine with respiratory disorders was also confirmed in the Republic of Korea, with a 10% prevalence at the herd level (22). Although present in swine populations in many countries, its influence on the development of respiratory disease in this animal is still unknown.

Respiratory diseases of infectious aetiology represent a significant economic issue in pig farming, and the combinations of pathogens responsible for their development are highly variable (19). In the past, research into the infectious causes of respiratory diseases in pigs in Serbia was mostly based on identifying common respiratory pathogens, such as *Pasteurella multocida, Actinobacillus pleuropneumoniae,* porcine circovirus 2, porcine reproductive and respiratory syndrome virus, swine influenza A virus (SIV) and Aujeszky’s disease virus (24, 28). In recent years, attention has also been given to respiratory pathogens that usually cause mild clinical outcomes (porcine circovirus 3, porcine respiratory coronavirus and porcine cytomegalovirus), but which can influence the aetiopathogenesis of porcine respiratory disease complex (PRDC) (7, 11, 27). Despite this and their documented worldwide presence in pig populations, research on PPIV1 and SOV in Serbia, to the best of our knowledge, has not yet been conducted. Therefore, the objectives of this research were to explore the presence of these two viruses in the Serbian swine population, determine the farm-level prevalence based on examination of samples originating from clinically diseased pigs and perform phylogenetic analysis of the detected strains.

## Material and Methods

**Sampling and sample preparation.** Samples for detection of PPIV1 and SOV were collected from pigs raised on commercial farms located in northern Serbia (Fig. 1). Four out of ten investigated farms were owned by the same company (Table 1). Sampling was conducted between October 2022 and November 2024 during surveillance of swine influenza. During farm visits, oral fluid samples (OF), nasal swabs (NS), and organ (lungs and lymph nodes) samples (OS) were collected from sick and dead nursery and fattening pigs. In total, 131 samples were collected (Table 1).

**Fig. 1.**
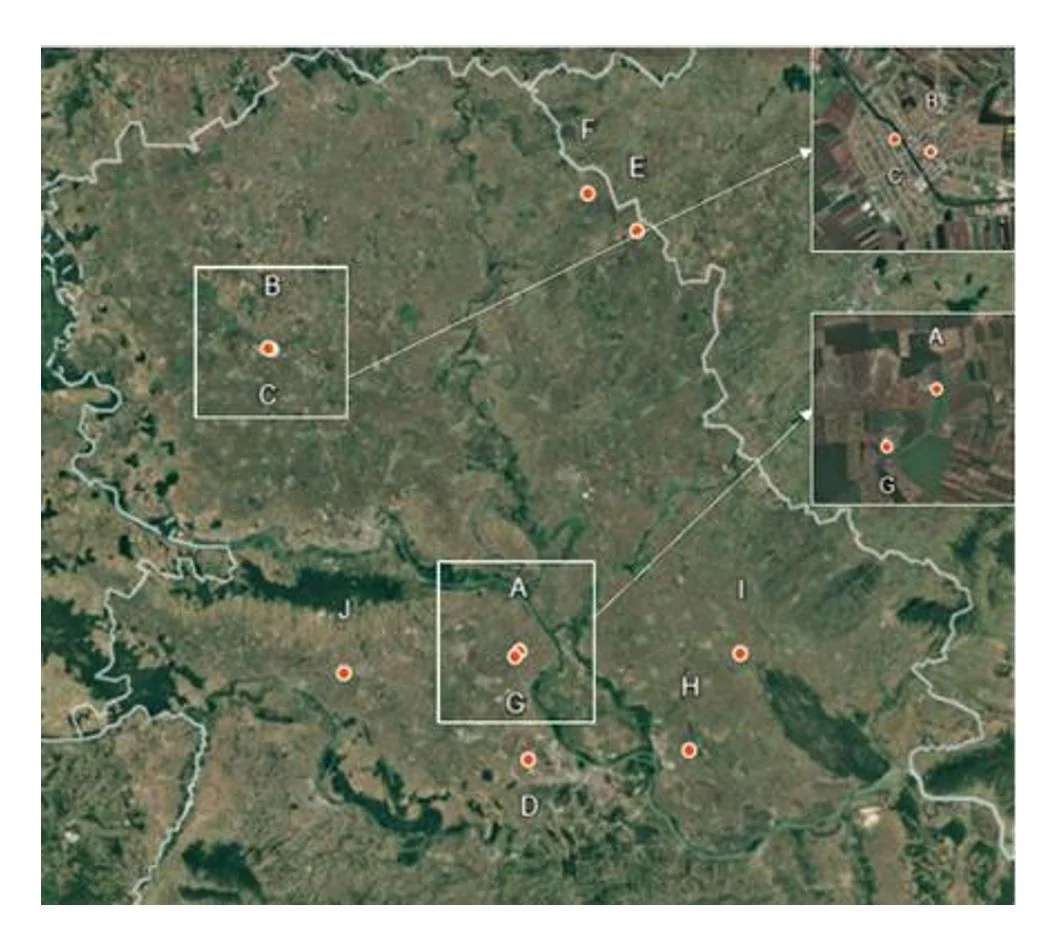
Geographical distribution of the commercial swine farms included in the study for detection of porcine parainfluenza virus 1, swine orthopneumovirus and swine influenza A virus. Farms are indicated by orange dots and capital letters (A–J)

**Table 1.** Characteristics of the investigated farms and number of collected pig samples per farm. The presence of porcine parainfluenza virus 1, swine orthopneumovirus and swine influenza A virus was assessed

Farm	Production type	Pig age group	OF	NS	OS
A*	multiplication farm	nursery	0	27	0
B	fattening farm	nursery	1	0	1
C	fattening farm	nursery	0	0	1
D	farrow-to-finish farm	nursery	1	10	0
E*	fattening farm	nursery	7	35	0
F	fattening farm	nursery	1	0	0
G*	farrow-to-finish farm	nursery	5	0	0
H	fattening farm	nursery	0	15	0
I*	farrow-to-finish farm	nursery	11	12	2
J	multiplication farm	nursery	2	0	0
Total sampled			28	99	4

* – farms belonging to the same owner; OF – oral fluid samples; NS – nasal swab samples; OS – organ samples comprising lung and lymph node tissue

Before testing, OF were centrifuged at 6,000 x *g* for 10 min to remove debris and other contaminants. Nasal swabs were soaked in 1 mL of PBS, and OS were homogenised with PBS in a 1 : 10 (w/v) ratio. After vigorous vortexing, all samples were centrifuged at 6,000 x *g* for 1 min. The resulting supernatants were collected and used for viral RNA extraction and subsequent PCR reactions.

**Extraction of RNA and PCR pathogen detection.** Extraction of viral RNA was performed using the IndiSpin Pathogen Kit (Indical Bioscience, Leipzig, Germany). Extracted RNA was eluted with 100 μL of Buffer AVE. As part of routine diagnostic examination, by requests of the farms’ owners, all samples were initially tested for SIV. Additionally, for research purposes, all samples were screened for PPIV1, and a subset of 77 samples (28 OF, 45 NS and 4 OS) was tested for SOV.

Swine influenza A virus (SIV) was detected using previously published primers and probes targeting a conserved region of the *M* (matrix) gene (4, 032). Screening for PPIV1 was performed using a real-time RT-PCR protocol for detection of the *HN* (haemagglutinin-neuraminidase) gene (17). Detection of SOV was carried out using an real-time RT-PCR protocol targeting the *M2* gene (22). Samples with a cycle threshold (Ct) below 40.0 were considered positive. All PPIV1-positive samples were further analysed by a gel-based RT-PCR using primers targeting the *F* (fusion) gene as described by Park *et al*. (23), which match portions of the GenBank reference sequence MT995732 (Table 2). All SOV-positive samples were additionally analysed using a gel-based RT-PCR with primers targeting the *G* (glycoprotein) gene (22). Further characterisation of detected SIVs was beyond the scope of this study.

**Table 2.** Porcine parainfluenza virus 1 *F* gene primer sequences

Primer	Reference sequence nucleotides	Primer sequence
F-For1	4801–4826	ACTTAGGTACAAGTTATCCAAAAA
F-For2	5537–5561	GAGAGAAGCTTAACATTACAGGC
F-Rev1	5611–5636	TCAATAATATCTGTATTCCCGATT
F-Rev2	6581–6603	TCTGCCACCTAAGTTTTTCTTA

For – forward; Rev – reverse

**Gene sequencing and phylogenetic analysis.** The amplicons obtained by conventional RT-PCR were purified and subjected to Sanger sequencing at the Faculty of Biology, University of Belgrade, Serbia. The obtained sequences were initially analysed using the Basic Local Alignment Search Tool (BLAST) against the National Center for Biotechnology Information (NCBI) database to confirm their viral identity. The list of sequences retrieved from NCBI was used for evolutionary analysis (Supplementary Tables S1 and S2). Sequence editing, alignment, and phylogenetic and evolutionary analyses of the PPIV1 *F* gene and SOV *G* gene were performed using MEGA version 7.0 software (13). Each genome segment was aligned separately using the ClustalW alignment tool (34). Phylogenetic trees were constructed using the maximum-likelihood method. Based on the corrected Akaike information criterion, the General Time-Reversible + gamma distribution + proportion of invariant sites model of evolution was selected and bootstrap analysis was performed with 1,000 replicates (20).

**Statistical analysis.** To assess association between sample type and the obtained results, Fisher’s exact test was used (30).

## Results

**Real-time RT-PCR results.** The real-time RT-PCR confirmed the circulation of at least one of the tested viruses on six farms (Fig. 2, Table 3). Co-circulation of two viruses was detected on four farms, while all investigated viruses were confirmed on one farm (Fig. 2, Table 3). In 3 out of 131 tested samples, SIV/PPIV1 (farm E), SIV/SOV and PPIV/SOV (farm I) coinfections were evident. None of the targeted viruses was detected on four farms (Fig. 2, Table 3). The majority of positive samples were OF (Table 3).

**Fig. 2.**
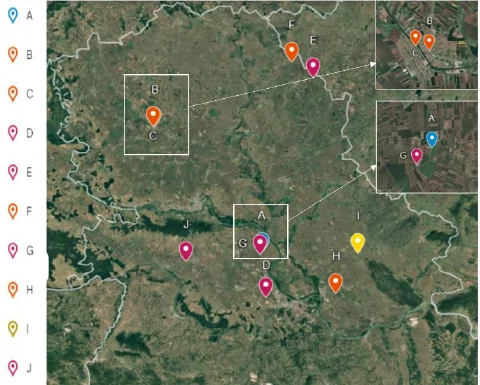
Distribution of farms with overall results of detection of porcine parainfluenza virus 1, swine orthopneumovirus and swine influenza A virus represented by different colours: blue pin – farm with one detected virus (farm A); pink pins – farms with two viruses detected (farms D, E, G and J); yellow pin – farm with three viruses detected (farm I); orange pins – farms where none of the targeted viruses was present in the tested samples (B, C, F and H)

**Table 3.** Results of real-time RT-PCR for detection of swine influenza A virus (SIV), porcine parainfluenza virus 1 (PPIV1) and swine orthopneumovirus (SOV) per farm

Farm	SIV T	SIV OS+	SIV OF+	SIV NS+	PPIV1 T	PPIV1 OS+	PPIV1 OF+	PPIV1 NS+	SOV T	SOV OS+	SOV OF+	SOV NS+
A	27	0	0	2	27	0	0	1	15	0	0	0
B	2	0	0	0	2	0	0	0	2	0	0	0
C	1	0	0	0	1	0	0	0	1	0	0	0
D	11	0	1	0	11	0	0	0	11	0	0	0
E	42	0	2	7	42	0	3	5	12	0	0	0
F	1	0	0	0	1	0	0	0	1	0	0	0
G	5	0	3	0	5	0	3	4	5	0	0	0
H	15	0	0	0	15	0	0	0	10	0	0	0
I	25	0	8	3	25	0	4	1	18	0	0	0
J	2	0	1	0	2	0	1	0	2	0	0	0
Total	131	0	15	12	131	0	12	6	77	0	0	0

T – tested; OS+ – positive organ samples comprising pig lung and lymph node tissue; OF+ – positive oral fluid samples; NS+ – positive pig nasal swabs

Analysis of the collected samples confirmed the circulation of SIV on six of the ten farms (60%). Overall, SIV was detected in 27 samples, comprising 15 OF and 12 NS (Table 3). Porcine parainfluenza virus 1 was detected on five farms, corresponding to a farm-level prevalence of 50%. On two of these farms, PPIV1 was identified in both OF and NS, whereas on the remaining farms the virus was detected exclusively in OF. Overall, 18 out of 131 tested samples were positive for PPIV1 (13.7%). Out of 28 OF, the virus was detected in 12 (42.9%), and out of 99 NS the virus was confirmed in 6 (6.1%) (Table 3). No virus was detected in OS. Cycle threshold values for PPIV1 ranged from 30.6 to 39.4. In 3 out of 42 tested samples originating from farm E, the genomes of both SIV and PPIV1 were detected, with lower Ct values for SIV (between 25.2 and 28.6). Using the described rRT-PCR protocol for SOV detection, two samples from farm I gave a positive result (Table 3), corresponding to a farm-level prevalence of 10%. One of these samples was also positive for SIV, while the other was positive for PPIV1. There was no statistically significant association between OF and NS samples for PPIV1 (P-value = 0.68) or SOV (P-value = 0.22).

**Gel-based RT-PCR results.** A conventional RT-PCR targeting the PPIV1 *F* gene was performed on 18 real-time RT-PCR-positive samples. Three nasal swab samples from the two farms (E and I) tested positive (Table 4). Additionally, two samples from farm I were positive for SOV (Table 4). All five PCR products obtained were subsequently subjected to Sanger sequencing.

**Table 4.** Results of gel-based RT-PCR for porcine parainfluenza virus 1 (PPIV1) and swine orthopneumovirus (SOV) per pig farm

Farm	PPIV1 OF T	PPIV1 OF P	PPIV1 NS T	PPIV1 NS P	Accession No.	SOV NS T	SOV NS P	Accession No.
D	1	0	0	0		0	0	
E	3	0	5	2	PX09864, PX098466	0	0	
G	4	0	0	0		0	0	
I	4	0	1	1	PX098465	2	2	PX09833, PX09834
J	1	0	0	0		0	0	
Total	12	0	6	3		2	2	

OF – oral fluid samples; NS – nasal swab samples; T – tested; P – positive

**Results of sequencing and phylogenetic analysis of PPIV1.** Sanger sequencing yielded only partial sequences of the PPIV1 F gene (lengths between 894 and 1020 base pairs). Comparison with sequences available in the NCBI database revealed that these three Serbian sequences shared the highest nucleotide identity (of approximately 95%) with PPIV1 strains from Italy (OR775101, sampled in 2023) and China (OK044758, sampled in 2019). The sequences have been deposited in GenBank under accession Nos PX098464, PX098465 and PX098466 (Table 4). Phylogenetic analysis showed that Serbian PPIV1 strains formed a distinct, well supported subclade (bootstrap value 99%) within clade 1, which also includes isolates from Europe and Asia. A separate clade comprised PPIV1 sequences from American and Asian strains (Fig. 3).

**Fig. 3.**
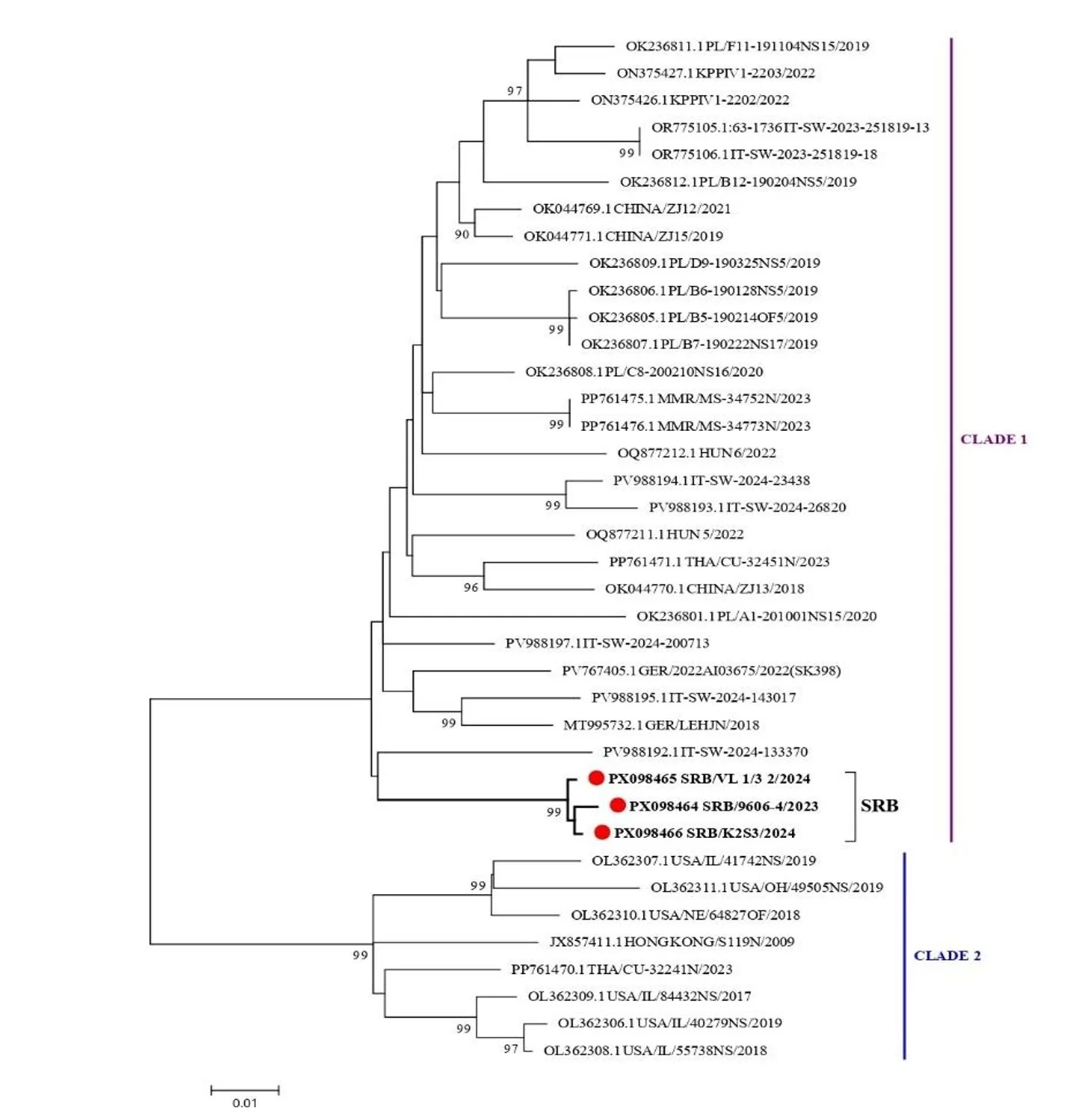
Phylogenetic analysis of the partial *F* gene of 38 nucleotide sequences of porcine parainfluenza virus 1 (PPIV1). Only bootstrap values higher than 75 are shown, and these are at nodes. All positions containing gaps and missing data were eliminated. Red dot – sequence obtained in this research; two-letter, six-digit code – GenBank accession number; slash-separated fields – geographic origin/isolate identifier/year

**Results of sequencing and phylogenetic analysis of SOV.** Sanger sequencing yielded only partial sequences of the G gene (619 and 632 base pairs). Comparison with sequences available in the NCBI database revealed the highest nucleotide identity (of approximately 92%) with a strain detected in the USA in 2012 (KX364383). The sequences have been deposited in GenBank under accession Nos PX098333 and PX098334 (Table 4). Alignment with other available SOV G gene sequences showed no insertions or deletions in the Serbian sequences. Phylogenetic analysis including pneumoviruses from swine and other host species revealed that the Serbian SOV strains clustered within a well-supported subclade of a larger clade containing swine, murine and canine strains, whereas human and bovine pneumoviruses formed an outgroup (Fig. 4).

**Fig. 4.**
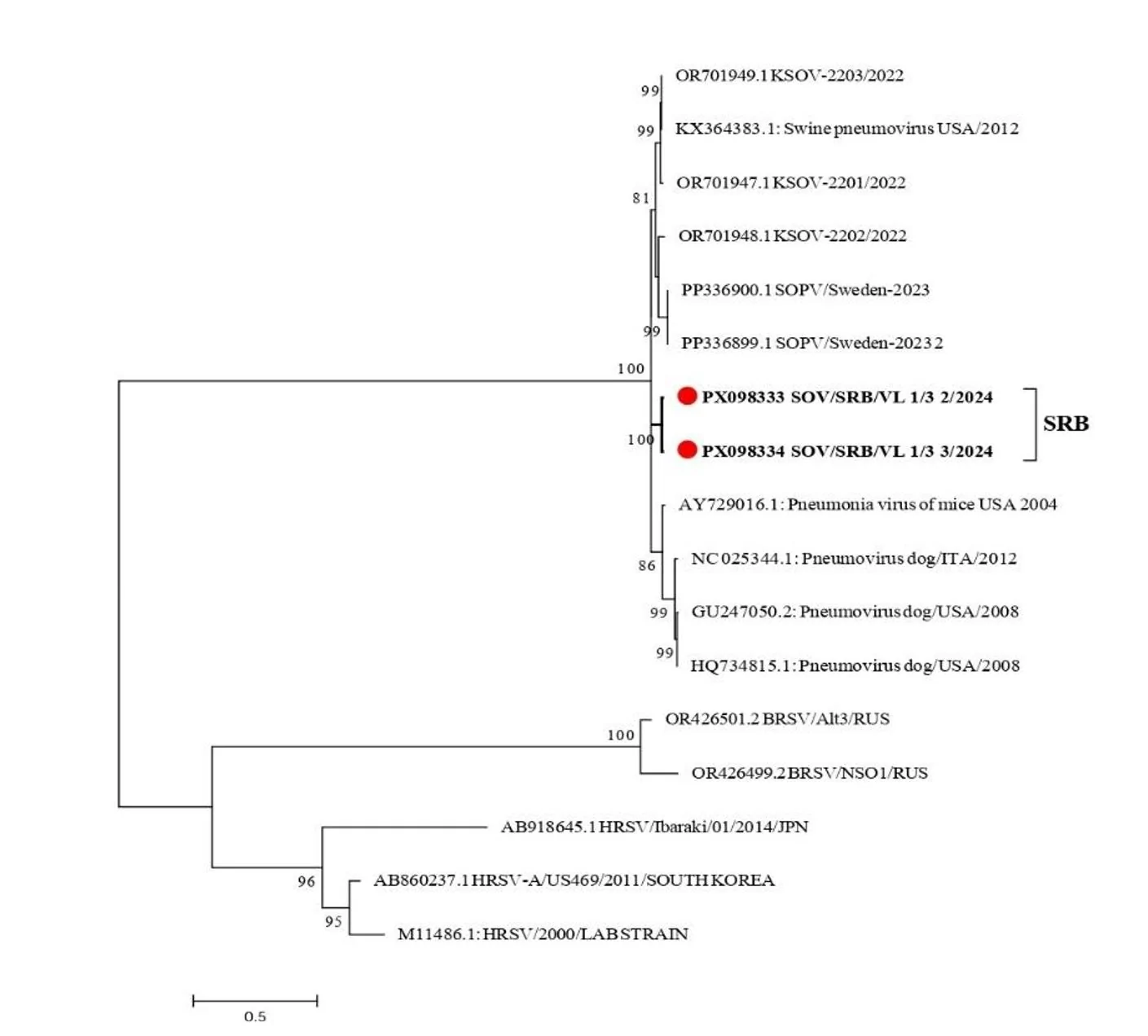
Phylogenetic analysis of the partial *G* gene of swine orthopneumovirus (SOV). Only bootstrap values higher than 75 are shown, and these are at nodes. All positions containing gaps and missing data were eliminated. Red dot – sequence obtained in this research; two-letter, six-digit code – GenBank accession number; slash-separated fields – *Orthopneumovirus* species/geographic origin/isolate identifier/year

## Discussion

The commercial pig farms included in this study are characterised by a high density of pigs, a short production cycle, introduction of live animals from other farms and multiple turnovers per year. Investigation of biosecurity practices on most of these farms revealed the need for improvements in their implementation (14). All these factors can increase the risk of the introduction and spread of infectious agents in a susceptible population. Noting evidence of the circulation of novel viral pathogens across the European pig sector, an investigation of PPIV1 and SOV on Serbian commercial pig farms was conducted. Swine influenza A virus is a pathogen commonly present on pig farms worldwide (5), including in Serbia (18). Its circulation on commercial farms in Serbia, combined with infections caused by other respiratory pathogens (18, 28), leads to a negative economic impact, which encourages farmers to report exacerbation of respiratory diseases and submit samples for testing. The results of SIV investigation confirm previous research, which found a high prevalence at the farm level (61.5%) (18). Based on the results of the OF and NS analyses, simultaneous circulation of SIV and PPIV1 was confirmed on four farms, while circulation of all three pathogens (SIV, PPIV1 and SOV) was confirmed on one farm. The presence of the new pathogens PPIV1 and SOV in SIV-infected populations has been documented in Germany, Spain and Chile (1, 8, 19), suggesting potential importance of these novel pathogens in PRDC pathogenesis. Confirmation of this condition is the finding of simultaneous infection with two pathogens (SIV+PPIV1, SIV+SOV or PPIV1+SOV) in three diseased animals from two farms. The detection of new respiratory viruses in sick pigs on Serbian farms increases the number of potential pathogen combinations that can be associated with disease (7, 11, 18, 24, 27, 28) and improves understanding of which pathogens to consider during PRDC laboratory examination.

The circulation of PPIV1 was confirmed on half of the tested farms. However, the study on three farms (B, C and F) was limited by very small sample sizes (fewer than five samples were tested) and may not fully reflect the epidemiological situation on these farms. In other European countries, the prevalence of PPIV1 at the farm level has been highly variable (3, 8, 10, 29, 31, 37), ranging from 18.8% in Denmark (3) to 76.7% in Poland (37). In our research, the viral genome was detected more frequently in OF than in NS. Similar results were reported by Stadejek *et al*. (33) and Park *et al*. (23).

Our findings confirm the value of OF for disease screening, as positivity in this type of sample can be detected even beyond the period of infectivity in the animals. Nevertheless, since oral fluid consists of unequal quantities of saliva, respiratory secretions, food components, microbiome and various cellular components from an imprecise number of animals, and is often contaminated with environmental dirt and non-target microorganisms, NS are more reliable samples for disease confirmation by molecular detection, downstream sequencing and phylogenetic analysis. Even so, Fisher’s exact test showed neither sample type to have an advantage over the other in determining prevalence at the farm level. When comparing the results of the real-time RT-PCR and gel-based RT-PCR, disagreement was evident. This discordance may be related to the low viral loads in the positive samples (Ct ≥ 30) and the lower diagnostic sensitivity of the later test.

The results of evolutionary analysis are consistent with previous findings on PPIV1 phylogeny, in which European and American strains were classified into two genetically distinct clades, while Chinese and other Asian isolates were distributed across both clades (31, 33, 38). The long branch lengths of Serbian strains indicate divergence from other previously characterised European strains.

Two of the sequenced strains originated from pigs raised on a fattening farm, while one was from a pig raised on a farrow-to-finish farm; both farms were owned by the same proprietor. This suggests the possibility of pathogen transmission associated with the exchange of animals within the regular production workflow.

Diagnostic examination for the presence of SOV revealed only two positive NS originating from nursery pigs from Farm I. Compared to other studies, the farm-level detection rate is the same as in the Republic of Korea (10%) (22), but significantly lower than the rate in SIV-positive farms in Spain (44%) (19). Based on partial *G* gene sequences, Serbian SOV strains were placed in a clade with swine, canine and murine *Orthopneumovirus* strains, which correlates with previously observed characteristics of this virus (9). Although our results are consistent with previously published findings, further research is necessary to improve diagnostic capacities and to advance genetic and molecular characterisation of circulating strains. The presence of this virus in samples from sick pigs suggests a possible influence on disease pathogenesis. However, although it has been studied in several pig populations (South Korea, Spain, Germany, USA) (8, 9, 19, 22), the role of this virus, either alone or in combination with other respiratory pathogens, in the development of respiratory disease in pigs has not yet been clarified.

## Conclusion

This is the first report of the circulation of PPIV1 and SOV on commercial pig farms in Serbia, as well as their presence in SIV-positive farms. Coinfections with SIV in nursery pigs on two farms suggest possible involvement of these viruses in the development of respiratory disease. Based on herd-level prevalence, we conclude that PPIV1 is a pathogen widely distributed on commercial pig farms in Serbia. Based on the partial *F* gene sequence, Serbian PPIV1 strains are grouped within clade 1, confirming their genetic relationship with previously characterised European strains. In contrast to PPIV1, SOV was detected on only one farm. Due to the limited availability of samples, further investigation is needed for more precise information regarding farm-level prevalence. The first partial *G* gene sequences of the strains circulating on the positive farm confirm these strains’ affiliation to the clade of other orthopneumoviruses. Wider exploration of the genome is necessary for more detailed insight into the evolution, genetic diversity and epidemiological significance of this virus in Serbian pig populations.

## Supplementary Material

Supplementary Material Details

Supplementary Material Details
