# Supplementary material for: First detection and phylogenetic analysis of porcine parainfluenza virus 1 and swine orthopneumovirus in swine reared on commercial farms in Serbia
Source: J Vet Res. 2026 Aug 24;70(3):353–61. doi: 10.2478/jvetres-2026-0048 (PMC13664715; doi:10.2478/jvetres-2026-0048)
Supplement: Supplementary file 1 [file jvetres-2026-0048_Supplementary_S1.pdf]

Supplementary Table S1. List of gene sequences obtained from the US National Center for Biotechnology Information used for construction of a porcine parainfluenza virus 1 *F* gene phylogenetic tree

GenBank accession No.	Year	Country of isolation
PV988197.1	2024	Italy
PV988195.1	2024	Italy
PV988194.1	2024	Italy
PV988193.1	2024	Italy
PV988192.1	2024	Italy
OR775106.1	2023	Italy
OR775105.1	2023	Italy
PP761476.1	2023	Myanmar
PP761475.1	2023	Myanmar
PP761471.1	2023	Thailand
PP761470.1	2023	Thailand
OQ877212.1	2022	Hungary
OQ877211.1	2022	Hungary
ON375427.1	2022	South Korea
ON375426.1	2022	South Korea
OL362311.1	2019	USA
OL362310.1	2018	USA
OL362309.1	2017	USA
OL362308.1	2018	USA
OL362307.1	2019	USA
OL362306.1	2019	USA
OK236812.1	2019	Poland
OK236811.1	2019	Poland
OK236809.1	2019	Poland
OK236808.1	2020	Poland
OK236807.1	2019	Poland
OK236806.1	2019	Poland
OK236805.1	2019	Poland
OK236801.1	2020	Poland
OK044771.1	2019	China
OK044770.1	2018	China
OK044769.1	2021	China
MT995732.1	2018	Germany
PV767405.1	2022	Germany
JX857411.1	2009	Hong Kong
