# Supplementary material for: First detection and phylogenetic analysis of porcine parainfluenza virus 1 and swine orthopneumovirus in swine reared on commercial farms in Serbia
Source: J Vet Res. 2026 Aug 24;70(3):353–61. doi: 10.2478/jvetres-2026-0048 (PMC13664715; doi:10.2478/jvetres-2026-0048)
Supplement: Supplementary file 2 [file jvetres-2026-0048_Supplementary_S2.pdf]

Supplementary Table S2. List of gene sequences obtained from the US National Center for Biotechnology Information used for construction of a swine orthopneumovirus (SOV) *G* gene phylogenetic tree

GenBank accession No.	Host species or virus species and year	Country of isolation
AY729016.1	Mouse 2004	USA
GU247050.2	Dog 2008	USA
HQ734815.1	Dog 2008	USA
KX364383.1	SOV 2022	USA
OR701949.1	SOV 2022	South Korea
OR701948.1	SOV 2022	South Korea
OR701947.1	SOV 2022	South Korea
NC025344.1	Dog 2012	Italy
PP336899.1	SOV 2022	Sweden
PP336900.1	SOV 2023	Sweden
OR426501.2	BRSV 2018	Russia
OR426499.2	BRSV 2019	Russia
AB918645.1	HRSV 2014	Japan
M11486.1	HRSV 2000	Unknown (laboratory strain)
AB860237.1	HRSV 2011	South Korea

BRSV – bovine respiratory syncytial virus; HRSV – human respiratory syncytial virus
